# Trace Amounts of Triple-Functional Additives Enable Reversible Aqueous Zinc-Ion Batteries from a Comprehensive Perspective

**DOI:** 10.1007/s40820-023-01050-4

**Published:** 2023-03-31

**Authors:** Ruwei Chen, Wei Zhang, Quanbo Huang, Chaohong Guan, Wei Zong, Yuhang Dai, Zijuan Du, Zhenyu Zhang, Jianwei Li, Fei Guo, Xuan Gao, Haobo Dong, Jiexin Zhu, Xiaohui Wang, Guanjie He

**Affiliations:** 1 Electrochemical Innovation Lab, Department of Chemical Engineering, University College London, London, WC1E 7JE UK; 2 State Key Laboratory of Pulp and Paper Engineering, South China University of Technology, Guangzhou, 510640 People’s Republic of China; 3 University of Michigan-Shanghai Jiao Tong University Joint Institute, Shanghai Jiao Tong University, Shanghai, 200240 People’s Republic of China

**Keywords:** Aqueous zinc-ion battery, Cationic shielding effect, Solid electrolyte interphase, pH value, Triple-functional additive

## Abstract

Although their cost-effectiveness and intrinsic safety, aqueous zinc-ion batteries suffer from notorious side reactions including hydrogen evolution reaction, Zn corrosion and passivation, and Zn dendrite formation on the anode. Despite numerous strategies to alleviate these side reactions have been demonstrated, they can only provide limited performance improvement from a single aspect. Herein, a triple-functional additive with trace amounts, ammonium hydroxide, was demonstrated to comprehensively protect zinc anodes. The results show that the shift of electrolyte pH from 4.1 to 5.2 lowers the HER potential and encourages the in situ formation of a uniform ZHS-based solid electrolyte interphase on Zn anodes. Moreover, cationic NH^4+^ can preferentially adsorb on the Zn anode surface to shield the “tip effect” and homogenize the electric field. Benefitting from this comprehensive protection, dendrite-free Zn deposition and highly reversible Zn plating/stripping behaviors were realized. Besides, improved electrochemical performances can also be achieved in Zn//MnO_2_ full cells by taking the advantages of this triple-functional additive. This work provides a new strategy for stabilizing Zn anodes from a comprehensive perspective.

**Supplementary Information:**

The online version contains supplementary material available at 10.1007/s40820-023-01050-4.

## Highlights

- A triple functional additive with a trace amount (1 mM) was proposed to protect Zn anodes.
- The additive lowers the hydrogen evolution reaction potential, encourages the formation of an in situ solid electrolyte interphase and shields the “tip effect”
- Dendrite free Zn deposition and highly reversible Zn plating/stripping behaviors were realized by the triple protections

**Supplementary Information:**

The online version contains supplementary material available at 10.1007/s40820-023-01050-4.

## Graphical abstract

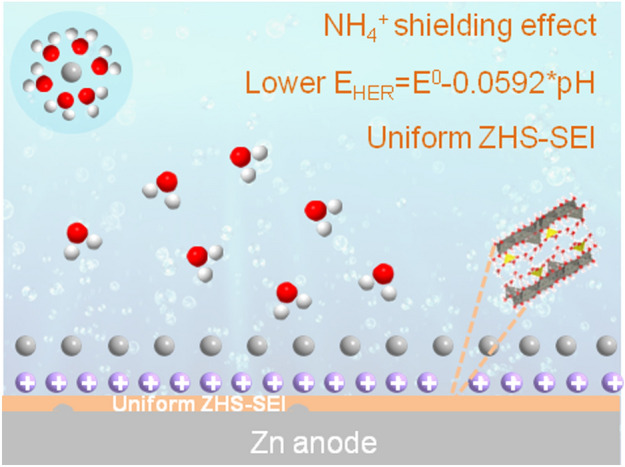

## Introduction

Lithium-ion batteries have dominated the energy storage field in the past four decades. However, the high cost caused by the shortage of lithium resources and the safety hazards caused by flammable organic electrolytes hinder their further grid-scale applications [1–4]. Aqueous zinc ion batteries are considered as the promising alternative battery technology for grid-scale energy storage applications in the post-lithium era due to their low cost, inherent safety, and high volumetric energy density of zinc anode (820 mAh g^−1^ and 5,855 mAh cm^−3^) [5–7]. Nevertheless, the implementation of this technology is still restricted by the dilemma of the notorious side reactions including hydrogen evolution reaction, Zn corrosion and passivation, and Zn dendrites [8–10].

An in-depth understanding of these issues is the cornerstone of achieving an effective remedy. Taking ZnSO_4_ electrolyte as an example, hydrogen evolution reaction (HER) is thermodynamically inevitable during the electrochemical processes due to the lower reduction potential of Zn^2+^/Zn (− 0.76 V vs. SHE) than that of HER (0 V vs. SHE) [11, 12]. The HER consumes H^+^ ions and causes an increase in the local OH^−^ ions concentration at the electrolyte/Zn anode interface. The production of OH^−^ ions will aggravate the formation of the loose Zn_4_SO_4_(OH)_6_·xH_2_O (ZHS) by-product, which leads to the corrosion and passivation of the Zn anode surface [13]. Correspondingly, such continuous corrosion and by-product formation will make an irregular electrode surface and uneven electric field and Zn^2+^ ion distribution. In turn, the inhomogeneous electric field and Zn^2+^ ion distribution will inevitably induce uneven Zn deposition and finally trigger the formation of the Zn dendrites under the “tip effect”, which probably pierce the separator and accelerate short-circuit [14]. These aspects are regarded as the main reasons for the poor reversibility and short lifespan, and how to solve these bottlenecks is of great research significance and application value.

Against this background, numerous strategies for alleviating these side reactions have been demonstrated. Among them, non-aqueous electrolytes and polymer gel electrolytes offer great opportunities to alleviate these notorious side reactions [15–17]. In addition, mixing additives into electrolytes is considered as a more promising strategy with regard to the facile process, cost-effectiveness, and broad availability [18, 19]. For instance, cationic additives such as TBA^+^, Li^+^, and Sc^3+^ were introduced into electrolytes, in which cations preferentially adsorbed on the Zn anode surface, providing a shielding effect to homogenize electric field and suppress dendrite growth [20–22]. Building an artificial solid electrolyte interphase (SEI) has been considered as another effective remedy to alleviate side reactions [23–27]. For example, various SEIs including COFs, poly(vinyl butyral), and zinc silicate have been coated on Zn anode surface to block the direct chemical or electrochemical reactions between Zn anode and electrolyte [28–30]. However, most additives can only provide a limited protection for zinc anode from a single aspect, and most ex-situ coated SEIs could be cracked and even detached from Zn anode during the cycling. Electrochemical reactions between electrolytes and anodes have been widely utilized to in situ construct SEIs during electrochemical cycling for Li-ion batteries. The in situ formed SEIs could not only seamlessly ensure cohesion of electrode but also the self-healable ability prevent the crack situation, which is essential for anode protection [11]. Unfortunately, given the much higher redox potentials of HER (0 V vs. SHE) and Zn^2+^/Zn (− 0.76 V vs. SHE) compared with that of Li + /Li (− 3.04 V vs. SHE), it is difficult to in situ form a reliable SEI before HER and Zn deposition [31, 32]. Therefore, multifunctional methodologies that can in situ build a reliable SEI and comprehensively protect zinc anode are highly desirable but are full of great challenges.

In this work, we demonstrate a triple-functional additive with trace amount (1 mM), ammonium hydroxide, into aqueous ZnSO_4_ electrolyte to comprehensively protect zinc anode. Firstly, in situ formation of a uniform ZHS-based SEI was encouraged by the shift of pH from 4.1 to 5.2, which can block the direct contact between water and Zn anode. In addition, the increased pH lowers the HER potential, relieving the HER tendency from the root. Moreover, cationic NH^4+^ preferentially adsorbed on the Zn anode surface and thus the “tip effect” was shielded and the electric field was homogenized. Consequently, the Zn//Zn symmetric cells show dendrite-free Zn deposition and highly reversible Zn plating/stripping behaviors. Besides, improved electrochemical performances can also be achieved in Zn//MnO_2_ full cells by taking the advantages of the triple-functional additive. This work provides a new strategy for stabilizing Zn anodes from a comprehensive perspective.

## Experimental Section

### Electrolyte and Electrode Preparation

2 M ZnSO_4_ electrolyte was prepared by dissolving 0.2 mol ZnSO_4_·7H_2_O (> 99%, VWR chemicals) into 100 mL deionized water with constant magnetic stirring for 30 min, which is denoted as BE. The designed electrolytes were then prepared by mixing proper amounts of 0.1 mol L^−1^ NH_3_·H_2_O with the as-prepared 2 M ZnSO_4_ solution to control the concentrations of NH_3_·H_2_O additives as 0.5, 1, 2, 3, and 5 mM. The optimized concentration of NH_3_·H_2_O is 1 mM and the corresponding electrolyte is denoted as DE. The MnO_2_ cathodes consisted of 70 wt% commercial MnO_2_ powder (precipitated active for synthesis, Sigma-Aldrich), 20 wt% acetylene black (battery grade, MTI), and 10 wt% poly(vinylidene fluoride) (PVDF; average Mw ~ 534,000, Sigma-Aldrich) with N-methyl-2-pyrrolidine (NMP; ≥ 99%, Sigma-Aldrich) as the solvent. Carbon paper (hydrophilic type; TORAY) was selected as the current collector. Polished Zinc foils with 1,000 mesh sandy paper (70 μm in thickness; ϕ12 mm circles) were used in Zn symmetric cells, Zn//Cu asymmetric cells, and Zn//MnO_2_ full cells.

### Materials Characterization

The XRD patterns were performed on a PANalytical Empyrean device with Cu Kα radiation. SEM was conducted on JEOL-JSM-6700F. A Bruker dimension Icon with Scanasyst device was employed to conduct AFM experiments. The in situ optical microscope was conducted on VisiScope® BL254 T1 (VWR) instrument. HANNA INSTRUMENTS HI9124 was performed to monitor the pH values.

### Electrochemical Measurements

The Zn//Zn symmetric cells, Zn//Cu cells, and Zn//MnO_2_ full cells were assembled based on CR2025 coin cell (316 stainless steel) with glass fiber (Whatman GF/D) as the separator. The evaluation of cyclic voltammetry, linear sweep voltammetry, chronoamperometry measurement, and electrochemical impedance spectroscopy tests were achieved by a VMP3 Biologic potentiostat. Linear polarization curves at 10 mV s^−1^ with Zn plate as the working electrode and Pt as the counter electrode and Ag/AgCl as the reference electrode. For Zn//MnO_2_ full cells, 0.1 M MnSO_4_ was added into the electrolyte to suppress Mn dissolution (2 M ZnSO_4_ + 0.1 M MnSO_4_ and 2 M ZnSO_4_ + 0.1 M MnSO_4_ + 1 mM NH_3_·H_2_O).

### Computational Details

All the calculations are performed in the framework of the density functional theory with the projector augmented plane-wave method, as implemented in the Vienna ab initio simulation package. The generalized gradient approximation proposed by Perdew-Burke-Ernzerhof (PBE) is selected for the exchange–correlation potential. The cut-off energy for plane wave is set to 480 eV. The energy criterion is set to 10^−6^ eV in iterative solution of the Kohn–Sham equation. All the structures are relaxed until the residual forces on the atoms have declined to less than 0.05 eV Å^−1^. The Zn diffusion barrier in the system is explored using nudged-elastic band (NEB) method.

## Results and Discussion

### Characterization of the Triple-functional Additives

**Fig. 1:**
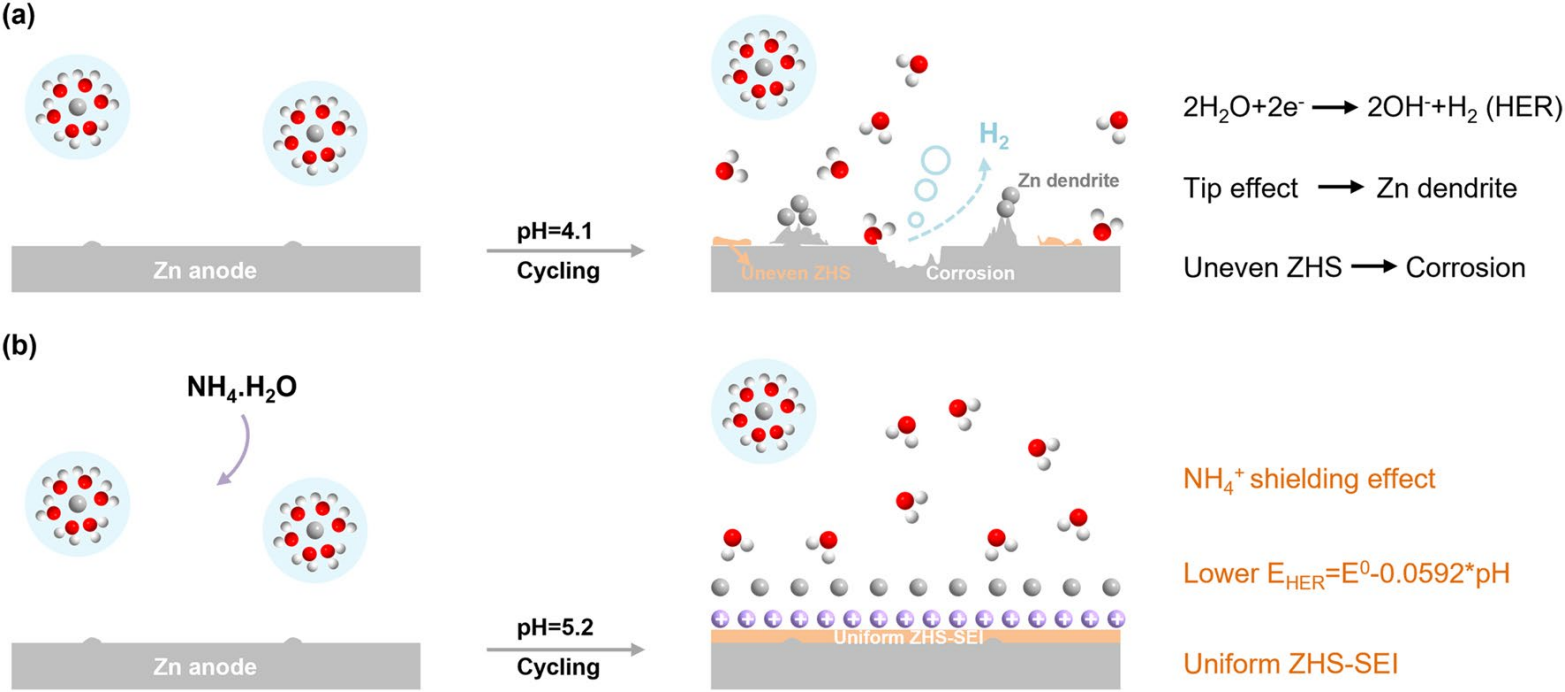
Schematic illustration of the Zn plating process in** a** BE and **b** DE

Zn metal is thermodynamically unstable in bare 2 M ZnSO_4_ electrolyte (BE, pH = 4.1). HER accompanied by the increased local pH value leads to self-corrosion and formation of ZHS. However, the as-formed ZHS from side reactions is generally randomly distributed on the Zn surface and cannot form a uniform SEI to block water [33]. HER, corrosion, and Zn dendrites would take place continuously on Zn/electrolyte interface (Fig. 1a) [34]. Theoretically, HER and ZHS formation are significantly affected by the pH value of the electrolyte according to the following reactions [35]:

1 $$2{\mathrm{H}}_{2}\mathrm{O}+2{\mathrm{e}}^{-}\to 2{\mathrm{OH}}^{-}+{\mathrm{H}}_{2} \left(\mathrm{HER}\right) $$

2 $${\mathrm{E}}_{\mathrm{HER}}={\mathrm{E}}^{0}-0.0592*\mathrm{pH }$$

3 $$4{\mathrm{Zn}}^{2+}+{{\mathrm{SO}}_{4}}^{2-}+6{\mathrm{OH}}^{-}+{\mathrm{xH}}_{2}\mathrm{O}\to {\mathrm{Zn}}_{4}{\mathrm{SO}}_{4}(\mathrm{OH}{)}_{6}.{\mathrm{xH}}_{2}\mathrm{O }$$

**Fig. 2:**
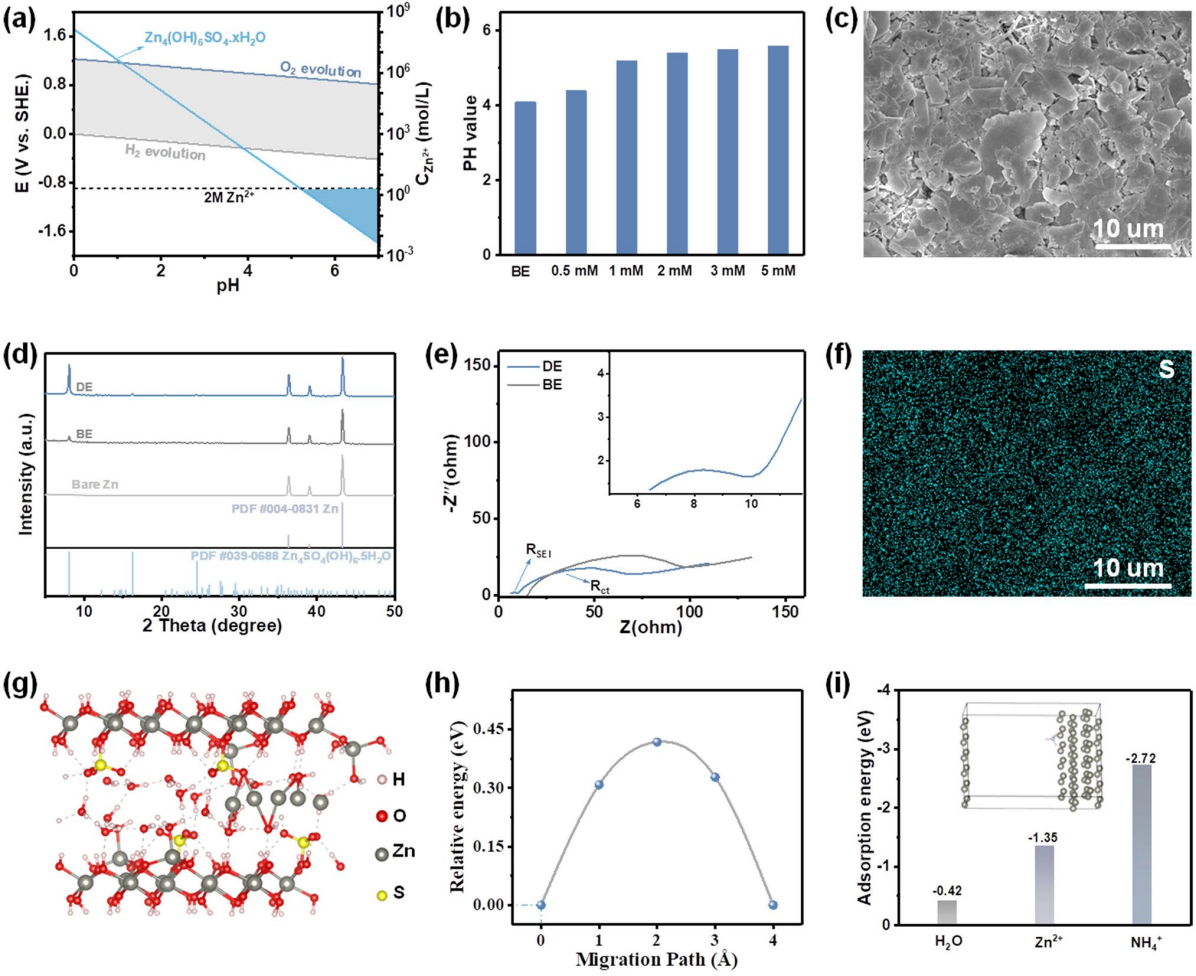
**a** Pourbaix diagram of the HER and ZHS formation. **b** pH values and hydrogen evolution potentials in different electrolytes. **c** SEM image of the Zn anode after immersed in DE for 8 h. **d** XRD patterns of Zn anode after immersed in different electrolytes for 8 h. **e** EIS spectra of Zn//Zn symmetric cells after cycled in different electrolytes. **f** Corresponding EDX mapping of the Zn anode after immersed in DE for 8 h. **g** DFT calculations of the typical Zn^2+^-diffusion path in ZHS. **h** Corresponding diffusion energy barriers. **i** The adsorption energy between H_2_O/Zn^2+^/NH_4_^+^ on the Zn (101) surface

In the neutral/mild acidic electrolyte, HER deeply depends on the pH of the electrolyte. Increasing the pH value would decrease the HER potential, which means that the HER is less likely to occur (Fig. 2a, Eqs. 1 and 2). On the other hand, increasing the pH value would increase the OH^−^ concentration. When the critical solubility OH^−^ is reached, ZHS will appear according to the *K*_sp_ (Eq. 3) [36]. Therefore, the higher pH value of the electrolyte, the more probably the ZHS will form (Fig. 2a).

Based on the above considerations, different amounts of ammonium hydroxide were added into BE. The pH value shifted quickly from 4.1 to 5.2 when the concentration of ammonium hydroxide increased to 1 mM (Fig. 2b). The increased pH can suppress the HER to a certain extent according to the Nernst equation (Fig. S1, Eq. 2). However, further increasing the concentration would cause the electrolyte precipitate (Fig. S2). Therefore, ZnSO_4_ electrolyte with 1 mM ammonium hydroxide was chose for further discussion (DE). Specially, a dense and uniform layer was formed on the Zn surface after immersed in DE for 8 h (Figs. 2c and S3). The corresponding EDX mapping image shows the uniform distribution of Zn, S, and O elements (Figs. 2f and S4). As shown in Fig. 2d, obvious peaks indexed to ZHS were observed after Zn plate immersed in DE for 8 h, indicating the successful formation of a uniform ZHS-based SEI on Zn anode (Fig. 2d). On the contrary, only randomly distributed ZHS was generated on Zn surface after immersed in BE for 8 h (Figs. S5–S6). In addition, electrochemical impedance spectroscopy (EIS) of Zn//Zn symmetric cells displays very different behavior in these two electrolytes. Only the Zn//Zn symmetric cell in DE shows a depressed semicircle assigned to SEI resistance (*R*_SEI_) at high frequency region (Fig. 2e) [37]. Moreover, the charge transfer resistance of Zn//Zn symmetric cell in DE is significantly smaller than that in BE, further proving the successful formation of the uniform ZHS-based SEI and thus preventing Zn from side reactions. Therefore, *in situ* formation of the uniform ZHS-based SEI was encouraged by the increased pH value.

The role of the as-formed ZHS-based SEI was further investigated by density functional theory (DFT) calculation. Figure 2g shows the possible diffusion channel in the tunnel-like framework of ZHS. The corresponding diffusion energy barrier is calculated to be 0.42 eV, suggesting its fast Zn^2+^ migration kinetics, which is very significant for a SEI (Fig. 2h) [38, 39]. Besides computational results, larger Zn^2+^ transference number and smaller nucleation overpotentials further confirm the fast Zn^2+^ migration kinetics and zincophilicity of the ZHS-based SEI (Figs. S7-S8). In addition, the adsorption energies of different species in electrolyte system on the Zn (101) surface were calculated. As shown in Fig. 2i, the adsorption energy of NH_4_^+^ (− 2.72 eV) is much lower than those of Zn^2+^ (− 1.35 eV) and H_2_O (− 0.42 eV), indicating that NH_4_^+^ is preferred to be absorbed on the surface of Zn electrode to shield the “tip effect” and homogenize the electric field [40]. Hence, by taking advantage of the comprehensive protection of the decreased HER potential, uniform ZHS-based SEI, and the shielding effect of NH_4_^+^, dendrite-free Zn deposition and highly reversible Zn plating/stripping behaviors are predictable for zinc-ion batteries in DE.

### Enhancements in Zn Anode Stability and Reversibility

**Fig. 3:**
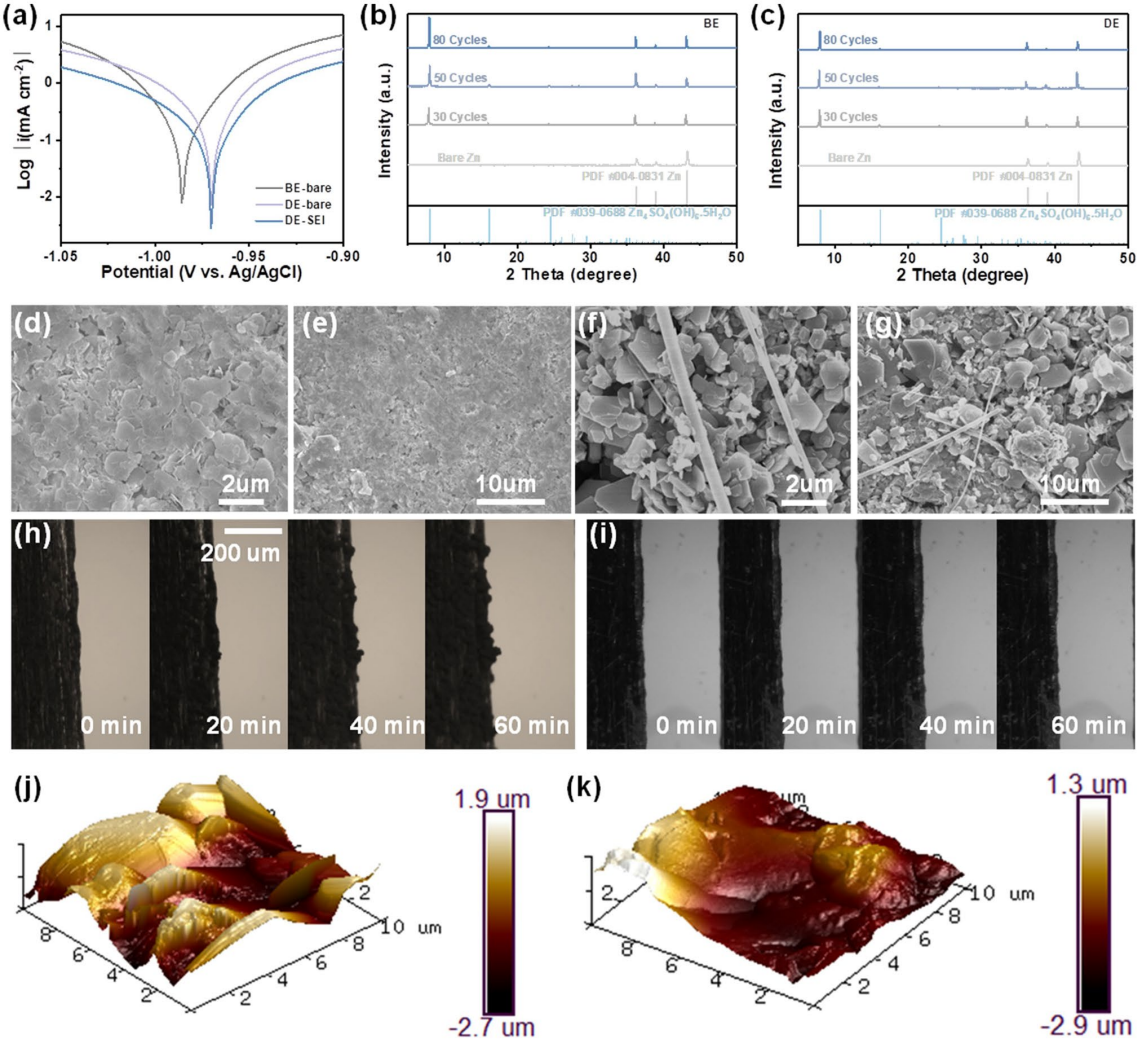
**a** Linear polarization curves. **b** XRD patterns of Zn anode after cycled in BE. **c** XRD patterns of Zn anode after cycled in DE. **d–e** SEM images of Zn anode after cycled in DE at high and low magnifications, respectively. **f–g** SEM images of Zn anode after cycled in BE at high and low magnifications, respectively. **h** *In situ* observation of Zn plating in the Zn//Zn cell with an BE. **i** *In situ* observation of Zn plating in the Zn//Zn cell with an DE. **j** AFM image of the Zn anode surface after cycled in BE. **k** AFM image of the Zn anode surface after cycled in DE

Linear polarization curves of different electrolytes were performed in a three-electrode configuration by using bare-Zn or SEI-Zn as the working electrode, Pt as the counter electrode, and Ag/AgCl as the reference electrode (Fig. 3a). Compared with the BE, the corrosion potential of bare Zn in the DE increases from − 0.99 to − 0.97 V and the corrosion current decreases from − 2.11 to − 2.15 mA cm^−2^, which demonstrate the reduced tendency toward corrosion reaction and corrosion rate due to the lower HER potential and the shielding effect of NH_4_^+^ [41]. Compared with the bare Zn, the corrosion current of SEI-Zn in DE decreases from − 2.15 to − 2.55 mA cm^−2^, suggesting the reduced corrosion rate with the help of the SEI [42]. Figure 3b–c records the XRD evolution of the cycled Zn electrodes harvested from Zn//Zn symmetric cells in BE and DE, respectively. The peaks indexed to ZHS emerges after 30 cycles and then dramatically strengthens after 80 cycles in BE, indicating continuous side reactions between BE and Zn (Fig. 3b) [30]. In contrast, the peaks related to ZHS remains stable after 80 cycles in DE, suggesting the formation of a robust ZHS-based SEI and the significantly suppressed side reactions (Fig. 3c) [43]. Moreover, the relatively steady EIS curves of Zn//Zn symmetric cells with increased cycles further prove the above conclusion (Fig. S9).

Scanning electron microscopy (SEM) were then carried out to probe how the electrolyte affects the Zn plating/stripping behaviors. The Zn electrode after cycled in DE exhibits a smooth surface with a dense SEI layer composed of polygonal ZHS flakes (Fig. 3d–e) [44, 45]. The SEI layer composed of polygonal flakes with a thickness of about 658 nm (Fig. S10). Moreover, the Zn anode surface still maintains a flat morphology even after 100 cycles, confirming the excellent stability of the SEI (Fig. S11). By comparison, the Zn electrode after cycled in BE shows a rough surface with obvious Zn dendrites, which is consistent with the XRD results (Fig. 3f–g). The same phenomenon can be found from the in situ optical microscopic observations. In the BE, random Zn protrusions start to form after 20 min plating and continuously grow into Zn dendrites after 40 min plating (Fig. 3h). On the contrary, the Zn surface remains flat without any Zn dendrites in the whole plating process in the DE (Fig. 3i). Moreover, atomic force microscopy (AFM) image also displays significantly reduced surface roughness of the cycled Zn electrode in the DE (Fig. 3j–k), further proving the dendrite-free Zn deposition.

**Fig. 4:**
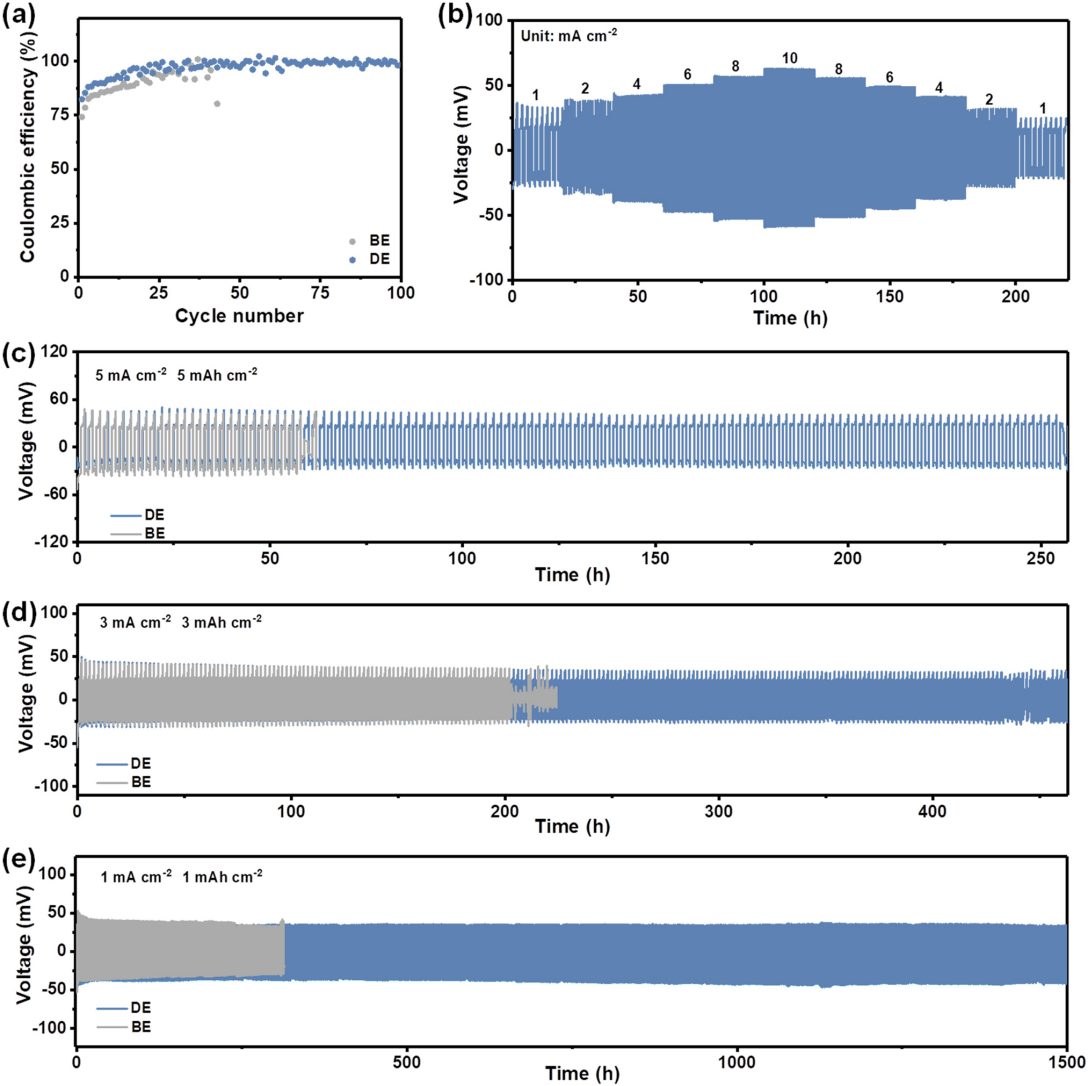
Zn plating/stripping behaviors in different electrolytes. **a** Coulombic efficiency of Zn//Cu asymmetric cells at 2 mA cm^−2^–1 mAh cm^−2^. **b** Rate performance of Zn//Zn symmetric cell in DE. **c-e** Long-term cycling performances of Zn//Zn symmetric cells at 5 mA cm^−2^–5 mAh cm^−2^, 3 mA cm^−2^–3 mAh cm^−2^, and 1 mA cm^−2^–1 mAh cm^−2^

As a result, dendrite-free Zn deposition with significantly suppressed side reactions can be achieved in DE by the comprehensive protection, which are beneficial to the reversibility and the stability of the Zn anode. The reversibility of Zn plating/stripping behaviors was evaluated in Zn//Cu half cells at a current density of 2 mA cm^−2^ and a capacity of 1 mAh cm^−2^. The Zn//Cu cell in BE only shows a limited lifespan of 43 cycles with relatively low Coulombic efficiencies, ascribed to the aforementioned dendrite growth and side reactions (Fig. 4a) [46]. In the case of the DE, the Zn//Cu cell exhibits a much longer lifespan and improved Coulombic efficiencies, demonstrating substantially suppressed side reactions and excellent reversibility [47]. Interestingly, only limitedly improved lifespan was achieved in the Zn//Cu cell when replaced ammonium hydroxide by (NH_4_)_2_SO_4_, suggesting the importance of the comprehensive protection in terms of decreased HER potential, uniform ZHS-based SEI, and the shielding effect of NH_4_^+^ (Fig. S12). The excellent cycling stability of Zn//Zn symmetric cells in DE under various current densities were also observed. The Zn//Zn symmetric cells using DE are able to stably cycle over 1,500 h and 450 h at 1 mA cm^−2^–1 mAh cm^−2^ and 3 mA cm^−2^–3 mAh cm^−2^, respectively (Fig. 4c-d). Even at a high current density of 5 mA cm^−2^ and capacity of 5 mAh cm^−2^, a longer cycle life over 250 h can also be maintained due to the comprehensive protection of Zn anode in DE, which dramatically surpasses the BE and (NH_4_)_2_SO_4_ counterparts (Figs. 4e and S13–S15). In addition to excellent stability and reversibility, the Zn//Zn symmetric cell also exhibits an excellent rate performance in DE (Fig. 4b). These results strongly support the fact that comprehensive Zn protection in DE leads to dendrite-free Zn deposition and highly reversible Zn plating/stripping behaviors.

### Electrochemical Performance of Full Cells

**Fig. 5:**
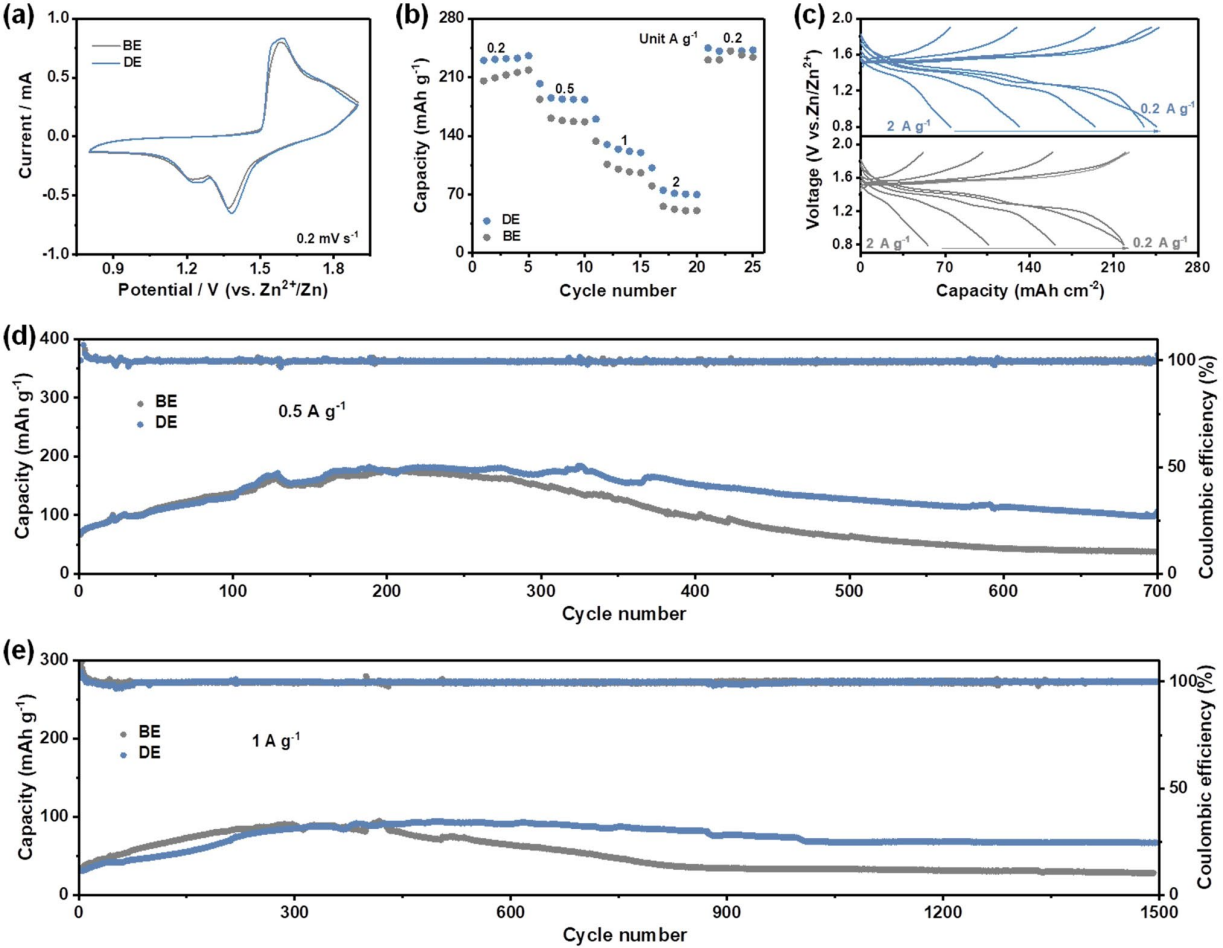
Electrochemical performances of Zn//MnO_2_ full cells. **a** Comparison of CV curves. **b** Comparison of rate performances. **c** Corresponding voltage profiles. **d** Cycling performance at 0.5 A g^−1^. **e** Cycling performance at 1 A g^−1^

To demonstrate the feasibility of the DE toward practical applications, full Zn-based full cells were assembled coupling with the commercial MnO_2_ cathode. As shown in Fig. S16, the diffraction peaks of the commercial MnO_2_ particles with an average size of several microns correspond to a γ-MnO_2_ phase [48]. Figure 5a shows the CV curves of the Zn//MnO_2_ full cells at a scan rate of 0.2 mV s^−1^ in different electrolytes. The Zn//MnO_2_ full cells exhibit similar redox peaks in BE and DE, indicating that the additive in DE would not affect the electrochemical charge storage mechanism of MnO_2_ [49–52]. Impressively, a better redox reaction kinetic behaviors were gained in DE electrolyte due to less polarization reflecting as relatively small potential differences between two pairs of redox peaks (Fig. 5a) [53, 54]. The rate performance of full cells using different electrolytes were illustrated in Fig. 4b, where the current density was raised stepwise from 0.2 to 2 A g^−1^ and returned to 0.2 A g^−1^. A better rate performance was achieved in DE, which further proving the better redox reaction kinetic behaviors in DE (Fig. 5c) [55, 56]. Figure 5d displays the comparison of long cycling stability evaluations in different electrolytes at 0.5 A g^−1^, in which a very low capacity retention performance of 55% was delivered along with a small specific capacity of 38.3 mAh g^−1^ after 700 cycles in BE. The rapid capacity decay is mainly attributed to severe side reactions occurring simultaneously at both anode and cathode sides during cycling [57]. Similarly, only a very small capacity of 28.3 mAh g^−1^ was retained in BE after 1,500 cycles in BE at a current density of 1 A g^−1^ (Fig. 5e). In contrast, higher specific capacities of 98.8 and 67.1 mAh g^−1^ were achieved along with relatively higher capacity retention performances after 700 and 1,500 cycles in DE at 0.5 and 1 A g^−1^, respectively. Therefore, improved electrochemical performances can also be achieved in Zn//MnO_2_ full cells by taking the advantages of the triple-functional additive.

## Conclusion

In this work, a triple-functional additive with trace amounts (1 mM), ammonium hydroxide, was demonstrated to protect Zn anode. The shift of electrolyte pH from 4.1 to 5.2 after introduced additive encourages the *in situ* formation of a uniform ZHS-based SEI on Zn anode while lowers the HER potential. Moreover, both experimental and theoretical calculations revealed that NH_4_^+^ is preferred to be absorbed on the surface of Zn anode to shield the “tip effect” and homogenize the electric field. Therefore, comprehensive Zn anode protection from the perspectives of the HER inhibition, the in situ formed SEI, and the cationic shielding effect was simultaneously realized by this triple-functional additive. Benefitting from the comprehensive protection, dendrite-free Zn deposition and highly reversible Zn plating/stripping behaviors were realized. Accordingly, The Zn//Zn symmetric cells using additive can sustain long-term cycling over 1,500, 450, and 250 h at 1 mA cm^−2^–1 mAh cm^−2^, 3 mA cm^−2^–3 mAh cm^−2^, and 5 mA cm^−2^–5 mAh cm^−2^, respectively. The Zn//MnO_2_ full cells with the additive also exhibit much better cyclic stabilities at both 0.5 and 1 A g^−1^. This work provides a new strategy for stabilizing Zn anodes from a comprehensive perspective.

### Supplementary Information

Below is the link to the electronic supplementary material.

Supplementary file1 (PDF 1029 KB)
