# Supplementary material for: Trace Amounts of Triple-Functional Additives Enable Reversible Aqueous Zinc-Ion Batteries from a Comprehensive Perspective
Source: Nanomicro Lett. 2023 Mar 31;15:81. doi: 10.1007/s40820-023-01050-4 (PMC10066055; doi:10.1007/s40820-023-01050-4)
Supplement: Supplementary file 1 — Supplementary file1 (PDF 1029 KB) [file 40820_2023_1050_MOESM1_ESM.pdf]

Supporting Information for

Trace Amounts of Triple-Functional Additives Enable Reversible Aqueous Zinc-Ion Batteries from A Comprehensive Perspective

Ruwei Chen [1, 2, #], Wei Zhang [1, #], Quanbo Huang [2, #], Chaohong Guan [3], Wei Zong [1], Yuhang Dai [1], Zijuan Du [1], Zhenyu Zhang [1], Jianwei Li [1], Fei Guo [1], Xuan Gao [1], Haobo Dong [1], Jiexin Zhu [1], Xiaohui Wang [2, *], and Guanjie He [1,*]

[1] Electrochemical Innovation Lab, Department of Chemical Engineering, University College London, London WC1E 7JE, UK

[2] State Key Laboratory of Pulp and Paper Engineering, South China University of Technology, Guangzhou 510640, P. R. China

[3] University of Michigan-Shanghai Jiao Tong University Joint Institute, Shanghai Jiao Tong University, Shanghai 200240, P. R. China

[#] Ruwei Chen, Wei Zhang, and Quanbo Huang contributed equally to this work.

* Corresponding authors. E-mail: fewangxh@scut.edu.cn (X. Wang), g.he@ucl.ac.uk (G. He)

Supplementary Figures

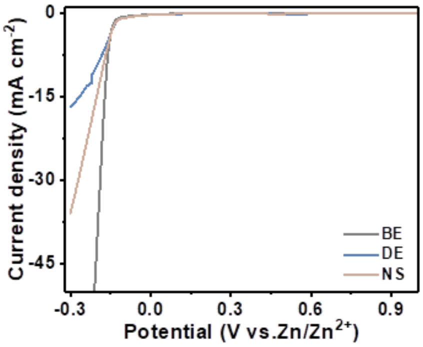

Fig. S1 LSV curves of Zn//Ti cells in different electrolytes

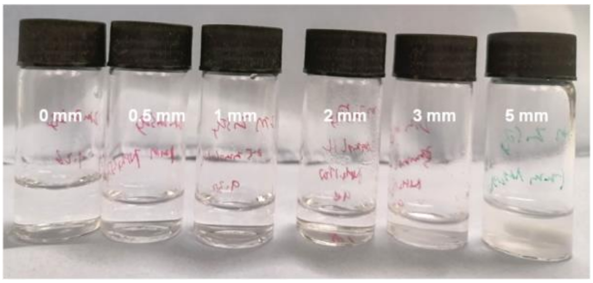

Fig. S2 Photograph of 2 M $ZnSO_4$ with different amounts of $NH_3 \cdot H_2O$

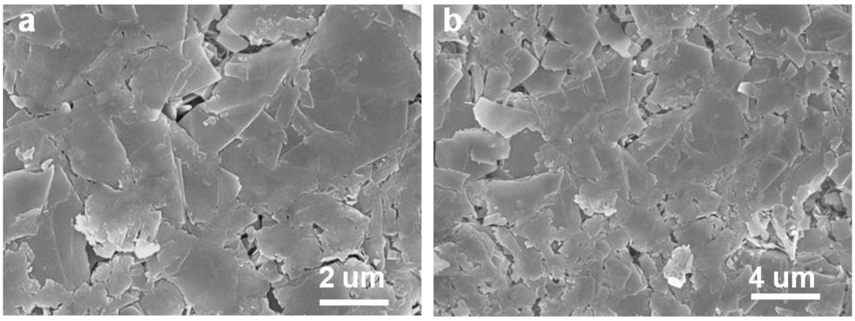

Fig. S3 SEM image of the Zn anode after immersing in DE for 8h at high and low magnifications, respectively

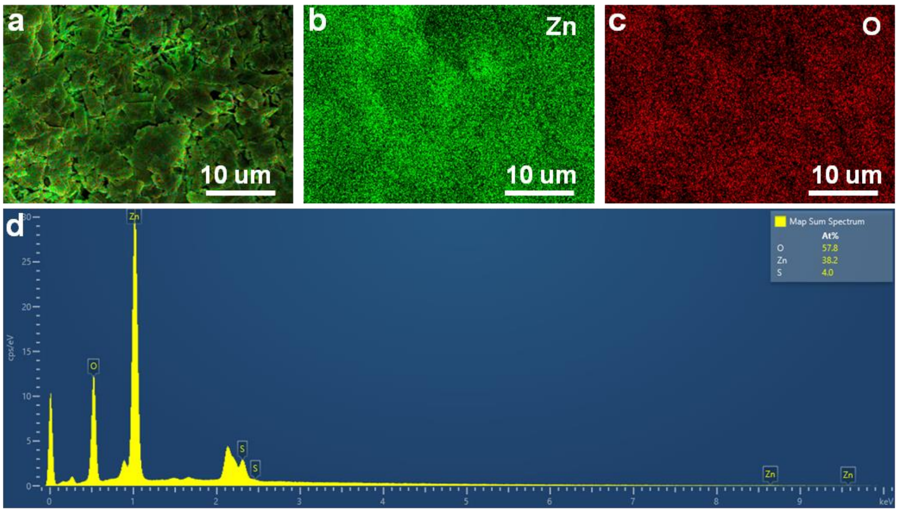

Fig. S4 (**a**) Overall EDX mapping of the Zn anode after immersing in DE for 8h. (**b**) Zn element distribution. (**c**) O element distribution. (**d**) Elements content spectrum

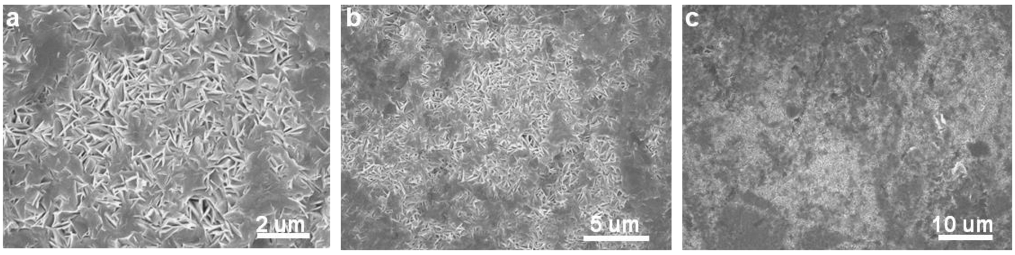

Fig. S5 (**a-c**) SEM image of the Zn anode after immersing in BE for 8h at high and low magnifications, respectively

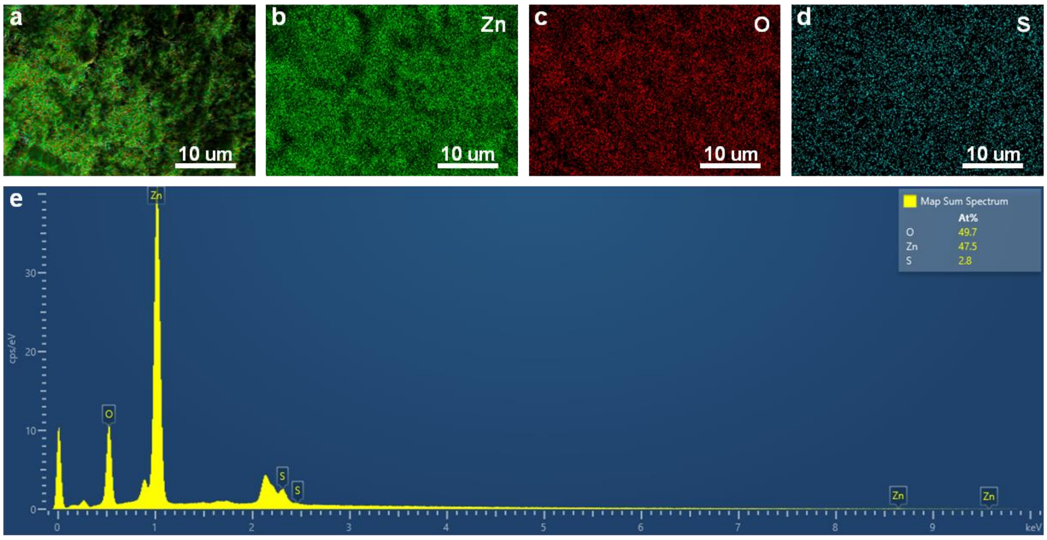

Fig. S6 EDX mapping of the Zn anode after immersed in BE for 8h. (**a**) Overall elements distribution. (**b**) Zn element distribution. (**c**) O element distribution. (**d**) S element distribution. (**e**) Elements content spectrum

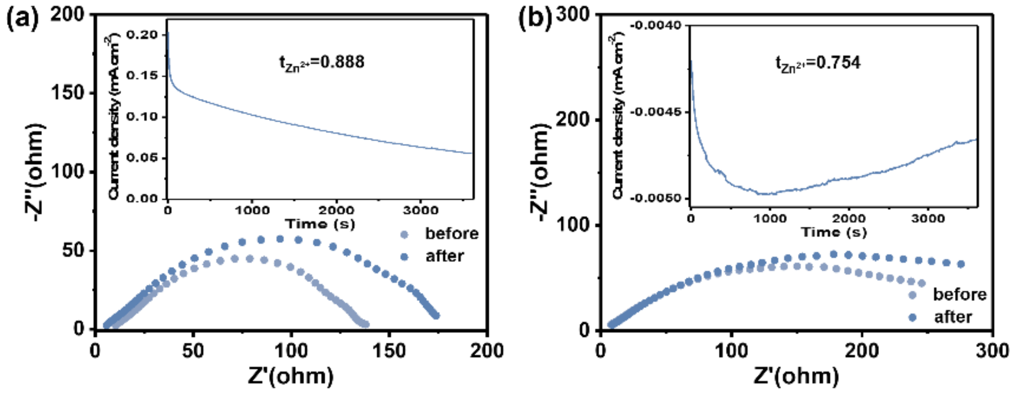

Fig. S7 EIS spectra of Zn//Zn symmetric cells before and after polarization. (**a**) DE. (**b**) BE

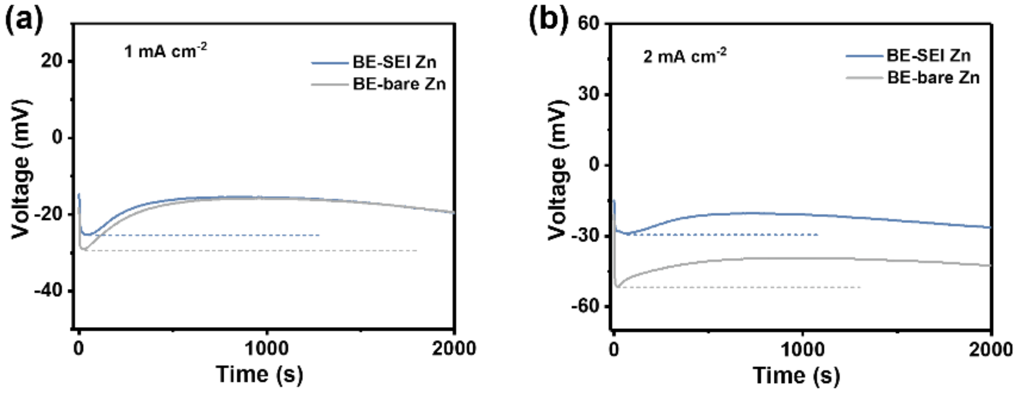

Fig. S8 Zn nucleation behavior on bare Zn and SEI Zn at different current densities. (**a**) 1 mA cm^{-2}. (**b**) 2 mA cm^{-2}

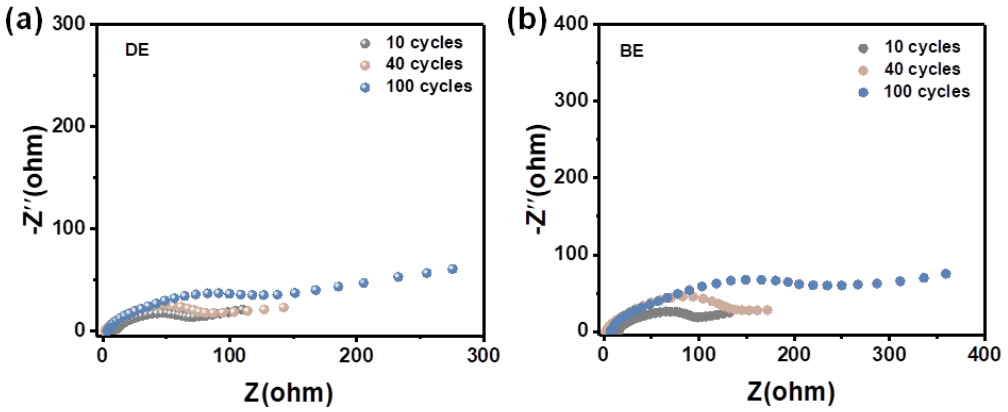

Fig. S9 (**a**) EIS curves of Zn//Zn symmetric cells in DE with different cycles. (**b**) EIS curves of Zn//Zn symmetric cells in BE with different cycles

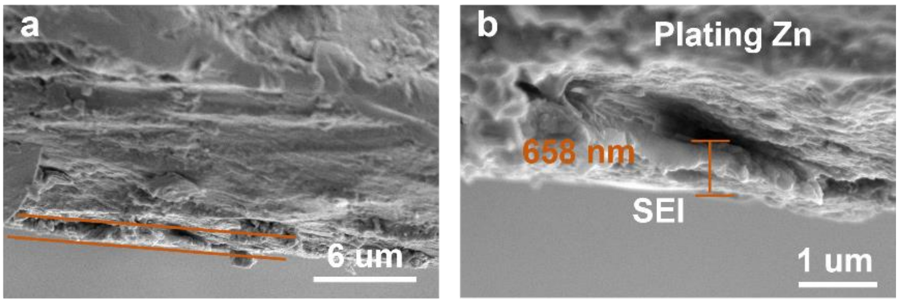

Fig. S10 (**a-b**) SEM images of the cross-section of Zn anodes after cycling in DE

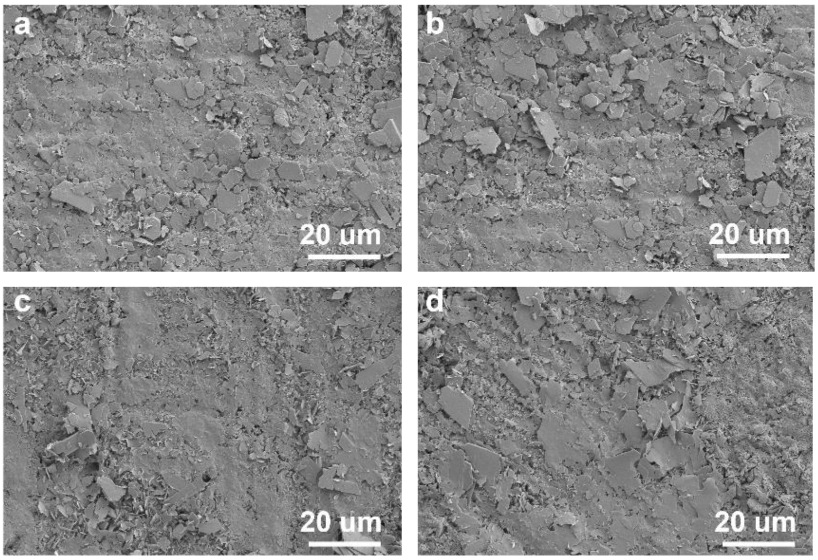

Fig. S11 SEM images of Zn anode surface after cycling in DE for (**a**) 30 cycles, (**b**) 50 cycles, (**c**) 80 cycles, and (**d**) 100 cycles

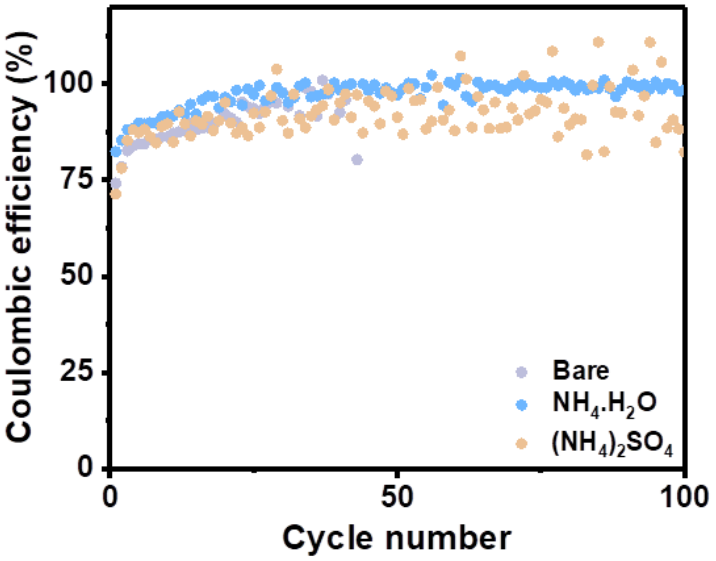

Fig. S12 Coulombic efficiency of Zn//Cu asymmetric cells in different electrolytes at 2 mA cm^{-2}-1 mAh cm^{-2}

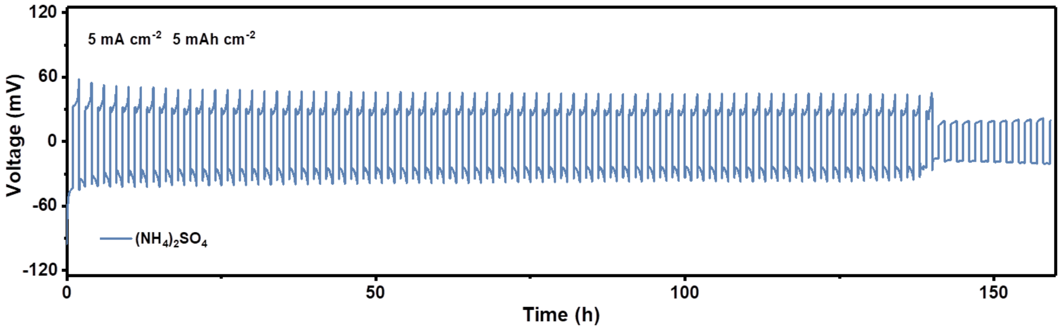

Fig. S13 Long-term cycling performances of Zn//Zn symmetric cell with $(NH_4)_2SO_4$ additive at 5 mA cm^{-2}-5 mAh cm^{-2}

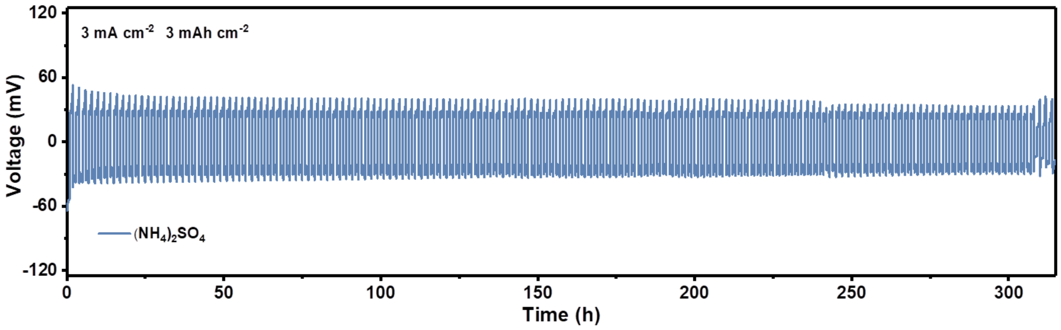

Fig. S14 Long-term cycling performances of Zn//Zn symmetric cell with $(NH_4)_2SO_4$ additive at 3 mA cm^{-2}-3 mAh cm^{-2}

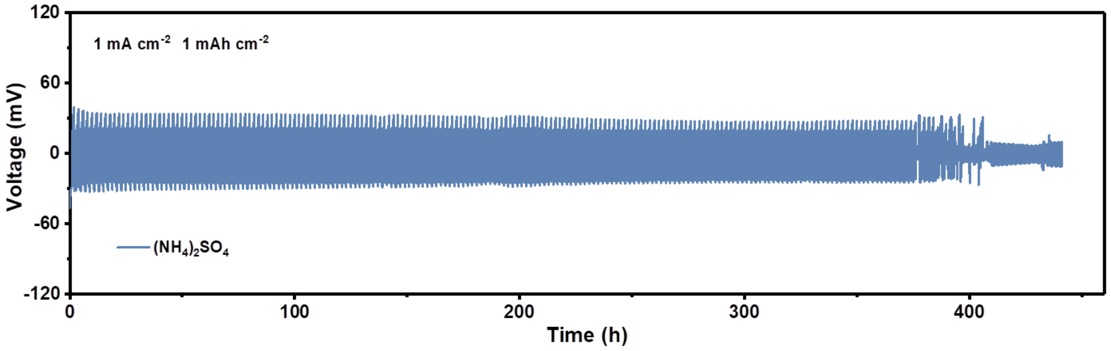

Fig. S15 Long-term cycling performances of Zn//Zn symmetric cell with $(NH_4)_2SO_4$ additive at 1 mA cm^{-2}-1 mAh cm^{-2}

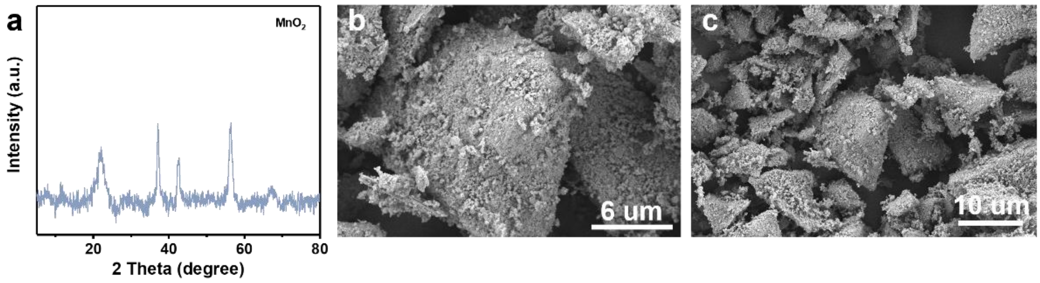

Fig. S16 (**a**) XRD patterns of commercial MnO_2. (**b-c**) SEM images of commercial MnO_2 at high and low magnifications, respectively
